# Prevalence of hypothyroidism in patients with hyponatremia: A retrospective cross-sectional study

**DOI:** 10.1371/journal.pone.0205687

**Published:** 2018-10-11

**Authors:** Takanobu Nagata, Shoko Nakajima, Atsushi Fujiya, Hiroshi Sobajima, Makoto Yamaguchi

**Affiliations:** 1 Department of Nephrology, Yokkaichi Municipal Hospital, Yokkaichi, Japan; 2 Department of Diabetology, Yokkaichi Municipal Hospital, Yokkaichi, Japan; 3 Department of Diabetology and Nephrology, Ogaki Municipal Hospital, Ogaki, Japan; Boston University School of Medicine, UNITED STATES

## Abstract

**Objective:**

Hypothyroidism has been suggested to be an uncommon cause of hyponatremia. However, little is known about the prevalence of hypothyroidism in patients with different levels of hyponatremia. The objective of this study was to investigate the prevalence of hypothyroidism among patients with hyponatremia of varying severity while taking into consideration potential confounders associated with thyroid function.

**Methods:**

All data on thyrotropin (TSH), free thyroxine (T4), and serum sodium (Na) levels were retrospectively collected from medical records at two Japanese tertiary hospitals. The main outcome measure was overt hypothyroidism, defined as TSH > 10.0 μIU/mL and free T4 < 1.01 ng/dL.

**Results:**

Of 71,817 patients, 964 patients (1.3%) had overt hypothyroidism. The prevalence of overt hypothyroidism in each category of hyponatremia (Na ≥136, 130–135, and ≤129 mEq/L) was 1.2% (787/65,051), 2.4% (124/5,254) and 3.5% (53/1,512), respectively. A significant increase in prevalence was observed as the severity of hyponatremia increased (P < 0.001 for trend). Multivariate logistic regression with adjustment for age, sex, kidney function, and serum albumin level showed that the odds ratios for overt hypothyroidism increased with increasing severity of hyponatremia when compared with Na ≥ 136 mEq/L (130–135 mEq/L: 1.43, 95% confidence interval [CI], 1.15 to 1.78, P = 0.001; ≤129 mEq/L: 1.87, 95% CI, 1.32 to 2.63, P < 0.001; P< 0.001 for trend).

**Conclusion:**

The prevalence of overt hypothyroidism was significantly higher as the severity of hyponatremia progressed, even after adjusting for potential confounders. Hypothyroidism should be differentiated in patients with hyponatremia.

## Introduction

Hypothyroidism is often referred to as a cause of hyponatremia, but several reports have shown that the association between thyroid function and serum sodium levels is very weak and of marginal clinical relevance[1–6]. Retrospective cross-sectional analyses have shown that the prevalence of hyponatremia and distribution of serum sodium levels were similar among euthyroid and hypothyroid patients[1]. A recent review and clinical practice guideline for hyponatremia have mentioned that even though hypothyroidism is one possible cause of hyponatremia, it should only be attributed to severe hypothyroidism, as in myxedema coma[4,6,7].

However, past reports had simply compared the prevalence of hyponatremia between patients with and without hypothyroidism[1,5,8]. In addition, some confounders such as sex, age, kidney function, and serum albumin level, which are reported to be associated with thyroid function[9–12], were not considered. Furthermore, no information was available about the prevalence of hypothyroidism with varying severity of hyponatremia.

The objective of the present retrospective cross-sectional study was to investigate the prevalence of hypothyroidism by severity of hyponatremia, and to clarify whether the association between the severity of hyponatremia and hypothyroidism is affected by confounding factors associated with thyroid function.

## Materials and methods

The study protocol was approved by the ethics committee of Yokkaichi Municipal Hospital and Ogaki Municipal Hospital. The study was conducted in accordance with the Declaration of Helsinki. The ethics committee approved waiver of informed consent for this study. The approval number of Yokkaichi Municipal Hospital was 2017–8 and that of Ogaki Municipal Hospital was 20161222–4.

All data on thyrotropin (TSH) levels between January 2008 and December 2017 were retrospectively collected from medical records at Yokkaichi Municipal Hospital in Yokkaichi, Japan, and Ogaki Municipal Hospital, in Ogaki, Japan. Both are tertiary hospitals in their respective medical districts. For each patient, initial TSH data during the study period and free thyroxine (T4) data from the same day were obtained. Patient age, sex, and levels of serum creatinine, blood urea nitrogen, potassium, chloride, total protein, albumin, and free triiodothyronine (T3) on the same day as TSH testing were also obtained. Next, we extracted the minimum serum sodium (Na) level within 3 days of TSH testing because TSH testing was unavailable at night and during holidays. Consequently, hyponatremia could have already been treated by the time of TSH testing. We excluded patients aged 17 years or younger.

The reference ranges for TSH and free T4 were 0.27 to 4.20 μIU/mL and 1.01 to 1.79 ng/dL, respectively. Estimated glomerular filtration rate (eGFR) was calculated using the equation from the Japanese Society of Nephrology: eGFR (mL/min/1.73 m^2^) = 194 × serum creatinine^-1.094^ × age^-0.287^ × 0.739 (for female patients)[13].

We defined hypothyroidism as TSH > 4.20 μIU/mL, and overt hypothyroidism as TSH > 10.0 μIU/mL plus free T4 < 1.01 ng/dL. Hyponatremia was defined as Na ≤ 135 mEq/L. Hyponatremia was classified as mild (130–135 mEq/L) or moderate to profound (≤129 mEq/L) according to guideline[14].

### Statistical analysis

Continuous variables were expressed as medians and interquartile ranges and compared using the Kruskal-Wallis test. Categorical variables were expressed as percentages and compared using Fisher’s exact test. Linear trends in proportions were assessed using the Cochran-Armitage test. Pearson’s correlation coefficients were used for correlation analysis. Univariate and multivariate logistic regression models were used to compute odds ratios and 95% confidence intervals (CIs) for hypothyroidism or overt hypothyroidism in each category of hyponatremia, with Na ≥ 136 mEq/L as the referent group. Multivariate models were adjusted for sex, age, eGFR, and serum albumin, which are factors that have previously been reported to be associated with thyroid function[9–12,15]. Univariate and multivariate linear trend tests were performed using each Na category as an ordinal variable.

All *P* values were two-tailed, and *P* < 0.05 was considered statistically significant. All statistical analyses were performed using EZR (Saitama Medical Center, Jichi Medical University, Saitama, Japan)[14], which is a graphical user interface for R (The R Foundation for Statistical Computing, Vienna, Austria).

## Results

Overall, data from 71,817 patients were included in this study. Patient characteristics are shown in Table 1. In total, 4,710 patients (6.6%) had hypothyroidism and 964 patients (1.3%) had overt hypothyroidism. The proportion of patients with hypothyroidism among those with a Na level of ≥136 mEq/L, 130–135 mEq/L, and ≤129 mEq/L was 6.1% (3,975 patients), 10.3% (539 patients), and 13.0% (196 patients), respectively. The proportion of overt hypothyroidism in the same categories was 1.2% (787 patients), 2.4% (124 patients), and 3.5% (53 patients), respectively.

**Table 1:** Patient characteristics by severity of hyponatremia.

		Serum sodium level			
Characteristic	All patients (n = 71,817)	≥136 mEq/L (n = 65,051)	130–135 mEq/L (n = 5,254)	≤129 mEq/L (n = 1,512)	*P*
Male sex, n (%)	34785 (48.4)	30927 (47.5)	3026 (57.6)	832 (55.0)	< 0.001
Age, years	66 [49, 76]	65 [48, 75]	72 [61, 81]	75 [65, 82]	< 0.001
Sodium, mEq/L	140 [138, 142]	140 [139, 142]	134 [132, 135]	126 [121, 128]	< 0.001
Potassium, mEq/L	4.2 [3.9, 4.4]	4.2 [3.9, 4.4]	4.2 [3.8, 4.6]	4.2 [3.8, 4.7]	< 0.001
Chloride, mEq/L	105 [103, 107]	105 [104, 107]	100 [97, 103]	93 [88, 97]	< 0.001
Blood urea nitrogen, mg/dL	14.9 [11.7, 19.3]	14.7 [11.7, 18.9]	16.9 [12.3, 25.2]	16.4 [11.1, 26.2]	< 0.001
Creatinine, mg/dL	0.71 [0.60, 0.90]	0.71 [0.60, 0.90]	0.80 [0.60, 1.11]	0.70 [0.52, 1.10]	< 0.001
eGFR, mL/min/1.73m^2^	74.0 [57.8, 90.7]	74.4 [58.8, 90.7]	69.2 [45.9, 88.9]	72.6 [46.4, 96.9]	< 0.001
Total protein, g/dL	7.10 [6.60, 7.50]	7.10 [6.70, 7.50]	6.60 [6.00, 7.30]	6.40 [5.70, 7.10]	< 0.001
Albumin, g/dL	4.20 [3.79, 4.50]	4.26 [3.88, 4.50]	3.50 [3.00, 4.00]	3.37 [2.77, 3.99]	< 0.001
TSH, μIU/mL	1.33 [0.83, 2.15]	1.33 [0.84, 2.12]	1.40 [0.78, 2.41]	1.46 [0.82, 2.69]	< 0.001
Free T3, pg/mL	2.82 [2.42, 3.20]	2.86 [2.47, 3.23]	2.40 [1.91, 2.84]	2.10 [1.68, 2.56]	< 0.001
Free T4, ng/dL	1.06 [0.94, 1.19]	1.05 [0.94, 1.18]	1.06 [0.92, 1.23]	1.13 [0.94, 1.32]	< 0.001

Categorical variables are expressed as the number (%) and continuous variables are expressed as the median [interquartile range]

eGFR, estimated glomerular filtration rate; TSH, thyrotropin; T3, triiodothyronine; T4, thyroxine.

**Fig 1:**
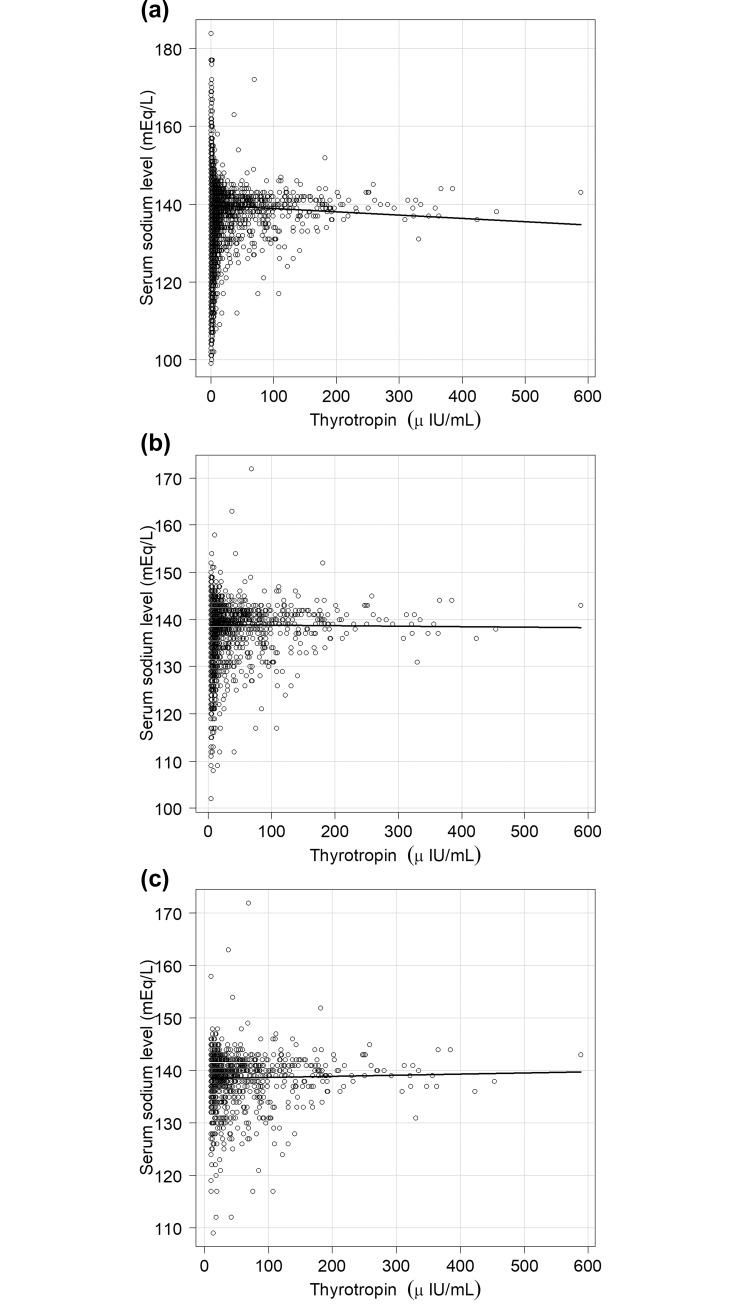
**Correlation between thyrotropin and serum sodium levels in all patients (a), patients with hypothyroidism (b), and overt hypothyroidism (c).** Pearson’s correlation coefficient: a, -0.021 (95% confidence interval [CI], -0.028 to -0.013, *P* < 0.001); b, -0.007 (95% CI, -0.035 to 0.021, *P* = 0.624); c, 0.029 (95% CI, -0.034 to 0.092, *P* = 0.365). Hypothyroidism was defined as TSH > 4.20 μIU/mL. Overt hypothyroidism was defined as TSH > 10.0 μIU/mL plus free T4 < 1.01 ng/dL.

The Cochran-Armitage trend test showed a significant increase in the proportion of patients with hypothyroidism and overt hypothyroidism as the severity of hyponatremia increased (both *P* < 0.001). The Pearson’s correlation coefficient for TSH and Na for the entire study population was -0.021 (95% CI, -0.028 to -0.013, *P* < 0.001) (Fig 1). Among patients with hypothyroidism and overt hypothyroidism, the values were -0.007 (95% CI, -0.035 to 0.021, *P* = 0.624) and 0.029 (95% CI, -0.034 to 0.092, *P* = 0.365) (Fig 1). A statistically significant but weak correlation was observed between TSH and Na in the entire study population. No statistically significant relationship was observed between TSH and Na among patients with hypothyroidism and overt hypothyroidism, respectively.

The unadjusted and adjusted odds ratios for hypothyroidism and overt hypothyroidism in each hyponatremia category are shown in Table 2. The adjusted odds ratios for hypothyroidism among patients with Na of 130–135 mEq/L and ≤129 mEq/L compared with Na ≥136 mEq/L were 1.11 (95% CI, 0.99 to 1.24, *P* = 0.054) and 1.32 (95% CI, 1.11 to 1.58, *P* = 0.003), respectively. The odds ratios for overt hypothyroidism were 1.43 (95% CI, 1.15 to 1.78, *P* = 0.001) and 1.87 (95% CI, 1.32 to 2.63, *P* < 0.001). A significant trend of increase in adjusted odds ratios was observed among patients with both hypothyroidism and overt hypothyroidism (*P* < 0.001 for trend).

**Table 2:** Associations between hyponatremia category and hypothyroidism.

		Serum sodium level			
Characteristic	Overall (n = 71,817)	≥136 mEq/L (n = 65,051)	130–135 mEq/L (n = 5,254)	≤129 mEq/L (n = 1,512)	*P* for trend
Hypothyroidism, n (%)	4710 (6.6)	3975 (6.1)	539 (10.3)	196 (13.0)	
	Unadjusted	Referent	1.75 (1.59–1.93)	2.28 (1.95–2.67)	< 0.001
	Model 1	Referent	1.42 (1.29–1.57)	1.77 (1.50–2.09)	< 0.001
	Model 2	Referent	1.11 (0.99–1.24)	1.32 (1.11–1.58)	< 0.001
Overt hypothyroidism, n (%)	964 (1.3)	787 (1.2)	124 (2.4)	53 (3.5)	
	Unadjusted	Referent	1.97 (1.61–2.38)	2.96 (2.19–3.94)	< 0.001
	Model 1	Referent	1.54 (1.26–1.89)	2.37 (1.74–3.21)	< 0.001
	Model 2	Referent	1.43 (1.15–1.78)	1.87 (1.32–2.63)	< 0.001

Hypothyroidism was defined as thyrotropin > 4.20 μIU/mL.

Overt hypothyroidism was defined as thyrotropin > 10.0 μIU/mL and free thyroxine < 1.01 ng/dL.

Model 1 adjusted for age, sex, and estimated glomerular filtration rate.

Model 2 adjusted for variables in Model 1 and serum albumin level.

## Discussion

This retrospective cross-sectional study investigated the prevalence of hypothyroidism in patients with hyponatremia. The correlation between TSH and Na levels was very weak, but the prevalence of hypothyroidism and overt hypothyroidism were significantly higher among patients with hyponatremia compared with patients with Na ≥ 136 mEq/L. Furthermore, a significant trend of increase in the prevalence of hypothyroidism and overt hypothyroidism was observed as the severity of hyponatremia increased, even after adjusting for potential confounders associated with thyroid function.

Past studies have reported a weak association between TSH and Na levels. Wolf et al. reported a very mild positive correlation between TSH and Na levels (-0.022; *P* = 0.046) in patients with newly diagnosed hypothyroidism in a single-center retrospective analysis[5]. They concluded that this weak association was not clinically significant. Schwarz et al. reported that TSH and Na levels were not significantly correlated (-0.02; *P* > 0.05) in patients whose TSH and Na levels were examined in the emergency department in a single center retrospective analysis[8]. The correlation coefficients in the present study were similar. The reason for this weak association could be that most patients with hypothyroidism were normonatremic. Therefore, the correlation could be weakened because the majority of patients had normonatremia when TSH and Na levels were analyzed as continuous variables. However, we thought that it was necessary to investigate the prevalence of hypothyroidism in patients with hyponatremia to understand the direction of the causal relationship.

It has been reported that there was no significant difference in distribution of Na levels between hypothyroid patients and controls, but every 10 mU/L rise in TSH is associated with a 0.14 mmol/L fall in Na[2]. Consequently, a recent review and clinical practice guideline for hyponatremia suggested that hypothyroidism rarely causes hyponatremia and hyponatremia should not be attributed to hypothyroidism except in cases of severe hypothyroidism, such as myxedema coma[4,6,7]. However, the present study showed that the prevalence of hypothyroidism and overt hypothyroidism in patients with hyponatremia tended to increase as the severity of hyponatremia increased. This result indicated that some patients with hyponatremia could have concomitant hypothyroidism.

This result seemed plausible given the pathophysiology of hyponatremia in patients with hypothyroidism. The putative mechanism by which hypothyroidism affects Na level is that hypothyroidism induces decreased cardiac output, which results in compensatory elevation of antidiuretic hormone levels and decreased GFR[16,17]. That may induce free water retention and decrease excretion by decreasing water delivery to the diluting segment of the nephron[6,18]. It is therefore reasonable that hypothyroidism could be a cause of hyponatremia, if not alone, even without severe hypothyroidism did not exist.

One strength of the present study was that confounders that could affect the prevalence of hypothyroidism were considered. An epidemiologic study revealed that hypothyroidism was more common in females and elderly individuals[10], and several reports have shown a higher prevalence of hypothyroidism in patients with chronic kidney disease[11,19]. In addition, it has been reported that serum albumin levels are significantly correlated with thyroid hormone levels[9]. Since the association between hyponatremia and hypothyroidism was significant even after adjusting for these factors, the results of present study can be considered more convincing.

This study has several limitations. First, this was a retrospective study. Since all TSH and free T4 testing in the present study was performed in patients who were suspected to have thyroid disorder by their attending physicians, the actual prevalence of hypothyroidism in patients with hyponatremia remains unknown. Second, patients had different comorbidities of various etiologies that could have caused hyponatremia, but patients’ medical records were not reviewed in this study. Third, information about the use of medications that could cause hyponatremia, such as diuretics, was not available because TSH testing occurred during the first visit for most patients. Thus, information about medications prescribed by other clinics could not be obtained. Forth, we could not investigate the causality between hypothyroidism and hyponatremia because of the cross-sectional study design. A prospective and longitudinal study is needed to clarify these issues.

## Conclusion

Although the correlation between TSH and Na was very weak, the prevalence of overt hypothyroidism increased significantly as the severity of hyponatremia progressed. Furthermore, the association remained even after adjusting for potential confounders associated with thyroid function. Thus, hypothyroidism should be differentiated in patients with hyponatremia.

## Supporting information

S1 Table Anonymous data set of 71817 patients. (CSV)

## References

[1] Croal BL, Blake AM, Johnston J, Glen AC, O’Reilly DS. Absence of relation between hyponatraemia and hypothyroidism. Lancet (London, England). 1997;350: 1402 10.1016/S0140-6736(05)65181-1 9365479

[2] Warner MH, Holding S, Kilpatrick ES. The effect of newly diagnosed hypothyroidism on serum sodium concentrations: A retrospective study [3]. Clin Endocrinol (Oxf). 2006;64: 598–599. 10.1111/j.1365-2265.2006.02489.x 16649984

[3] Pantalone K, Hatipoglu B. Hyponatremia and the Thyroid: Causality or Association? J Clin Med. 2014;4: 32–36. 10.3390/jcm4010032 26237016 PMC4470237

[4] Aylwin S, Burst V, Peri A, Runkle I, Thatcher N. ‘Dos and don’ts’ in the management of hyponatremia. Curr Med Res Opin. 2015;31: 1755–1761. 10.1185/03007995.2015.1072706 26173050

[5] Wolf P, Beiglböck H, Smaijs S, Wrba T, Rasoul-Rockenschaub S, Marculescu R, et al Hypothyroidism and Hyponatremia: Rather Coincidence Than Causality. Thyroid. 2017;27: 611–615. 10.1089/thy.2016.0597 28351291

[6] Liamis G, Filippatos TD, Liontos A, Elisaf MS. Hypothyroidism-associated hyponatremia: Mechanisms, implications and treatment. Eur J Endocrinol. 2017;176: R15–R20. 10.1530/EJE-16-0493 27484454

[7] Spasovski G, Vanholder R, Allolio B, Annane D, Ball S, Bichet D, et al Clinical practice guideline on diagnosis and treatment of hyponatraemia. Nephrol Dial Transplant. 2014;29: 1–39. 10.1093/ndt/gft315 24569496

[8] Schwarz C, Leichtle AB, Arampatzis S, Fiedler GM, Zimmermann H, Exadaktlyos AK, et al Thyroid function and serum electrolytes: Does an association really exist? Swiss Med Wkly. 2012;142 10.4414/smw.2012.13669 22987514

[9] Feinstein EI, Kaptein EM, Nicoloff JT, Massry SG. Thyroid function in patients with nephrotic syndrome and normal renal function. Am J Nephrol. 1982;2: 70–6. Available: http://www.ncbi.nlm.nih.gov/pubmed/7180903 10.1159/000166587 7180903

[10] Vanderpump MPJ, Tunbridge WMG, French JM, Appleton D, Bates D, Clark F, et al The incidence of thyroid disorders in the community: A twenty-year follow-up of the Whickham Survey. Clin Endocrinol (Oxf). 1995;43: 55–68. 10.1111/j.1365-2265.1995.tb01894.x 7641412

[11] Chonchol M, Lippi G, Salvagno G, Zoppini G, Muggeo M, Targher G. Prevalence of subclinical hypothyroidism in patients with chronic kidney disease. Clin J Am Soc Nephrol. 2008;3: 1296–1300. 10.2215/CJN.00800208 18550654 PMC2518789

[12] Åsvold BO, Bjøro T, Vatten LJ. Association of thyroid function with estimated glomerular filtration rate in a population-based study: The HUNT study. Eur J Endocrinol. 2011;164: 101–105. 10.1530/EJE-10-0705 20930062

[13] Matsuo S, Imai E, Horio M, Yasuda Y, Tomita K, Nitta K, et al Revised Equations for Estimated GFR From Serum Creatinine in Japan. Am J Kidney Dis. 2009;53: 982–992. 10.1053/j.ajkd.2008.12.034 19339088

[14] Kanda Y. Investigation of the freely available easy-to-use software “EZR” for medical statistics. Bone Marrow Transplant. 2013;48: 452–458. 10.1038/bmt.2012.244 23208313 PMC3590441

[15] Chaker L, Bianco AC, Jonklaas J, Peeters RP. Hypothyroidism. Lancet. 2017;390: 1550–1562. 10.1016/S0140-6736(17)30703-1 28336049 PMC6619426

[16] Skowsky WR, Kikuchi TA. The role of vasopressin in the impaired water excretion of myxedema. Am J Med. 1978;64: 613–621. 10.1016/0002-9343(78)90581-8 645727

[17] Hanna FWF, Scanlon MF. Hyponatraemia, hypothyroidism, and role of arginine-vasopressin. Lancet. 1997;350: 755–756. 10.1016/S0140-6736(05)62563-9 9297992

[18] Derubertis FR, Michelis MF, Bloom ME, Mintz DH, Field JB, Davis BB. Impaired water excretion in myxedema. Am J Med. 1971;51: 41–53. 10.1016/0002-9343(71)90322-6 5570319

[19] Rhee CM, Kalantar-Zadeh K, Streja E, Carrero J-J, Ma JZ, Lu JL, et al The relationship between thyroid function and estimated glomerular filtration rate in patients with chronic kidney disease. Nephrol Dial Transplant. 2015;30: 282–287. 10.1093/ndt/gfu303 25246335 PMC4309193

